# Network pharmacology and experiment validation investigate the potential mechanism of triptolide in oral squamous cell carcinoma

**DOI:** 10.3389/fphar.2023.1302059

**Published:** 2024-01-08

**Authors:** Puyu Hao, Pengcheng Zhang, Ying Liu, Yang Cao, Lianqun Du, Li Gao, Qingyang Dong

**Affiliations:** ^1^ Modern Research Center for Traditional Chinese Medicine, Shanxi University, Taiyuan, China; ^2^ Environmental and Operational Medicine Research Department, Academy of Military Medical Sciences, Academy of Military Sciences, Tianjin, China

**Keywords:** network pharmacology, molecular docking, oral squamous cell carcinoma, triptolide, JAK-STAT signaling pathway, MAPK signaling pathway

## Abstract

**Objective:** This study aimed to investigate the molecular mechanism of triptolide in the treatment of oral squamous cell carcinoma (OSCC) via network pharmacology and experimental validation.

**Methods:** The network pharmacological method was used to predict the key targets, detect the signal pathways for the treatment of OSCC, and screen the critical components and targets for molecular docking. Predicted targets were validated in cellular and xenograft mouse model.

**Results:** In this study, we predicted action on 17 relevant targets of OSCC by network pharmacology. PPI network demonstrated that Jun, MAPK8, TP53, STAT3, VEGFA, IL2, CXCR4, PTGS2, IL4 might be the critical targets of triptolide in the treatment of OSCC. These potential targets are mainly closely related to JAK-STAT and MAPK signaling pathways. The analysis of molecular docking showed that triptolide has high affinity with Jun, MAPK8 and TP53. Triptolide can suppress the growth of OSCC cells and xenograft mice tumor, and downregulate the expression of Jun, MAPK8, TP53, STAT3, VEGFA, IL2, CXCR4, PTGS2 to achieve the therapeutic effect of OSCC.

**Conclusion:** Through network pharmacological methods and experimental studies, we predicted and validated the potential targets and related pathways of triptolide for OSCC treatment. The results suggest that triptolide can inhibit the growth of OSCC via several key targets.

## 1 Introduction

OSCC is the most common type of oral malignant tumors, originating from the mucosal lining of the oral cavity and affecting the tongue, palate, mouth floor, alveolar ridge, and buccal mucosa (Meng et al., 2021). OSCC accounts for the majority of morbidity and mortality of head and neck squamous cell carcinoma (HNSCC), which is the sixth most common cancer in human (Shi et al., 2021; de Camargo et al., 2022). In 2020, 377,713 newly diagnosed cases and 177,757 deaths from lip and oral cavity cancers were reported worldwide. The traditional risk factors of OSCC include tobacco consumption and alcohol abuse. The habit of chewing betel quid additionally contributes to the development of OSCC in the Southeast Asia and Pacific regions, resulting in a high incidence of OSCC in these areas, such as India and Taiwan (Lin et al., 2020; Chamoli et al., 2021). However, owing to the lack of incipient symptoms and limited awareness of the risk factors among patients with OSCC, most of patients are diagnosed when OSCC has progressed further, resulting in a survival rate of only 34.9% among people with advanced OSCC (Wang et al., 2020). The mainstay treatment of OSCC is surgery, with or without adjuvant treatment (chemotherapy and radiotherapy), which is applied based on the histopathological characteristics of the resection (Almangush et al., 2020). However, multiple factors such as tumor invasion, lymph node metastasis, distant metastasis, and high recurrence rates result in a low 5-year survival rate only 50% among patients with OSCC. Most OSCC patients with distant metastasis, such as lung cancer, die within 1 year (Manzano-Moreno et al., 2021; Fan et al., 2022).

Triptolide is a natural diterpenoid epoxide that is obtained from *Tripterygium wilfordii* Hook F, a traditional Chinese medicinal herb also known as “Lei Gong Teng” or “Thunder God Vine” (Gang et al., 2022). Triptolide was isolated in 1972 for the first time and then used as an anti-leukemia drug (Kupchan et al., 1972). Triptolide shows considerable potential for clinical application in the treatment of many diseases, with various effects including anti-inflammatory, anti-rheumatic, immunosuppressive and anticancer effects (Gao et al., 2021). However, its narrow therapeutic window, poor solubility and low bioavailability, multiple organ toxicity, especially hepatoxicity, limit its clinical applications (Noel et al., 2019; Hu et al., 2022). The cell growth of oral cancer and HNSCC can be inhibited, which is related to a dose-dependent apoptosis induced by triptolide and its derivatives (Chen et al., 2009; Caicedo-Granados et al., 2014). Triptolide can also suppress the expression of programmed death ligand 1 (PD-L1), metastasis-associated protein 1 (MTA1) and certain cytokines, to repress the cell proliferation, invasion, angiogenesis, and migration in oral cancer (Yang et al., 2017; 2019; Kuo et al., 2021). However, the biological effect of triptolide on OSCC has not been fully understood from a comprehensive perspective.

Within this context, network pharmacology is a new method for filtering and predicting critical interactions between the chemical constituents of Chinese medicinal herbs and disease targets based on bioinformatics, systems biology, chemoinformatics and polypharmacology (Song et al., 2020). In this study, we explored the effects of triptolide in the treatment of OSCC *in vitro and in vivo*, using network pharmacology method to investigate the molecular biological mechanisms and develop a novel and effective therapeutic strategy for OSCC patients.

## 2 Materials and methods

### 2.1 Target genes prediction of triptolide and OSCC

#### 2.1.1 Prediction of OSCC-related target genes

We searched for OSCC-related targets within the GeneCards and Online Mendelian Inheritance in Man (OMIM) database, and set “oral squamous cell carcinoma” as the keyword. Top 310 genes with scores >50 were selected from the GeneCards database (Supplementary Table S1 and Supplementary Table S2). We integrated the results from the GeneCards and OMIM databases, deleted duplicates, and obtained the final OSCC-related target genes. The web addresses of the public databases are shown in Table 1.

**TABLE 1:** Basic information of the database used for the screening of triptolide in the treatment of OSCC.

Name	URL
OMIM	https://www.omim.org/
GeneCards	https://www.genecards.org/
SwissTargetPrediction	http://www.swisstargetprediction.ch/
Uniprot	https://www.uniprot.org
PubChem	https://pubchem.ncbi.nlm.nih.gov/
STRING	https://cn.string-db.org/
RCSB PDB	https://www.rcsb.org/
TCMSP	https://old.tcmsp-e.com/tcmsp.php
Bioinformatics	http://www.bioinformatics.com.cn/
Venny 2.1.0	https://bioinfogp.cnb.csic.es/tools/venny/index.html

#### 2.1.2 Prediction of genes targeted by triptolide

We got the targets related to triptolide from the Traditional Chinese Medicine Systems Pharmacology (TCMSP) and Swiss Target Prediction database, then transformed them into gene names via the Uniprot website. We also downloaded the chemical structure of triptolide from the PubChem website and obtained the target genes from SwissTargetPrediction.

### 2.2 Construction and analysis of component–target–disease network

We imported the selected targets of OSCC and triptolide into an online platform, bioinformatics, for Venn analysis and visualization, which we then imported into Cytoscape for component–target–disease network visualization. This visualization reflected the complex relationships among the three.

### 2.3 Screening and enrichment of core targets

We used the CytoNCA plug-in in Cytoscape to analyze the protein interaction networks and chose the top 10 targets as core target genes according to their betweenness values. Gene Ontology (GO) and Kyoto Encyclopedia of Genes and Genomes (KEGG) analyses of the core target genes were conducted via R software.

### 2.4 Macromolecular docking

We obtained the 3D protein crystal structures of the candidate targets of triptolide from the Protein Data Bank (PDB) database, which we subsequently imported into Maestro with the small molecular structures of triptolide obtained from the PubChem database for molecular docking simulations.

### 2.5 Cell lines and reagents

Human tongue squamous cell carcinoma cells lines SCC-25 and CAL-27 were used for our experiments. The above cells were purchased from Qingqi (Shanghai) Biotechnology Development Co., Ltd., and have been identified and tested for continuous *mycoplasma* contamination. Cells were fixed with 4% paraformaldehyde for 5 min, subsequently cleaned with PBS, then added DAPI dye, and observed after staining for 5 min. During the time of the experiments no obvious *mycoplasma* contamination was detected in cultured cells. The cells were cultured in high-glucose Dulbecco’s Modified Eagle Medium (DMEM) (Biosharp, BL304A) as well as 10% fetal bovine serum (FBS) (EVERY GREEN, 11011-8611) and 1% penicillin-streptomycin solution (Biosharp, BL505A). The parameters for cell culture were set as 37°C and 5% CO2. Triptolide was derived from Sichuan Weikeqi Biotechnology Co., Ltd. (WKQ-0000333, CAS: 38748-32-2). The stock solution was prepared by dissolving triptolide powder in dimethyl sulfoxide (DMSO), and diluted to certain concentrations in the following cell experiments. The final DMSO concentration in the medium was less than 0.1%.

### 2.6 Cell proliferation assay

Cells were inoculated in 96-well plates at 3000 cells per well and cultured for 24 h. Each experimental group had six parallel holes. In the next day we changed fresh medium with triptolide at 0, 0.01, 0.1, 1, 10, 100, 1,000 and 10000 nM for 24 h. We added 10 μL of Cell Counting Kit-8 (CCK-8) (Biosharp, BS350B) for each well. After incubating for 50 min, we measured the optical density (OD) at 450 nm on the multi-mode microplate readers (SpectraMax M5). We observed the results of CCK-8 with 6 technical replicates and 2 biological replicates.

### 2.7 Real-Time quantitative polymerase chain reaction (RT-qPCR)

We extracted total RNA by TRIzol reagent (Thermo Fisher Scientific, 15596026) and detected the purity of collected RNA via NanoDrop One Microvolume UV-Vis Spectrophotometers. 1 μg RNA was used to reverse-transcribe to cDNA using PrimeScript RT Master Mix (TaKaRa, RR036A) based on the specifications. The primers are listed in Supplementary Table S3. We mixed primers, cDNA and PowerUp SYBR Green Master Mix (Thermo Fisher Scientific, A25742) and conducted RT-qPCR on QuantStudio 5 Real-Time PCR System (Table 2). Finally we calculated relative gene expression by the 2^−ΔΔCT^ method.

**TABLE 2:** Sample Mixing System for RT-qPCR..

Reagent	Usage amount	Final concentration
5X PrimeScript RT Master Mix (Perfect Real Time)	2 μL	1X
Total RNA		
RNase Free dH2O	up to 10μ l	

### 2.8 Xenotransplantation of tumors

The specific-pathogen-free (SPF) male BALB/c nude mice (4-6 weeks old, 23.40 ± 1.61 g) with same age and genetic background, were acquired from Beijing Vital River Laboratory Animal Technology Co., Ltd. All animals were kept under standard laboratory conditions with free access to standard water and food. All procedures were conducted in accordance with the “Guiding Principles in the Care and Use of Animals” (China) and approved by Ethics Committee of Laboratory Animal Welfare, Institute of Environmental and Operational Medicine Research Department (LACUC of AMMS-04-2023-010). 1× 10^6^ SCC-25 cells were subcutaneously injected into each BALB/c nude mouse. When the tumors grew to 50 mm^3^, the mice were divided into two groups of 10 mice each. Based on previous studies and considering the solubility of triptolide, we determined the dosage and method of triptolide (Yu et al., 2020; Cai et al., 2021; Deng et al., 2021). 1) In the control group, the mice were intraperitoneally injected with 10% DMSO and corn oil. 2) In the triptolide group, the mice were intraperitoneally injected with 0.75 mg/kg triptolide (dissolved in DMSO and diluted with corn oil in a 1:9 ratio), once every 3 days for 3 weeks. We used the Vernier scale to measure tumor size. The formula for calculating tumor volume (mm^3^) was 0.5 × (shortest diameter)^2^ × (longest diameter).

### 2.9 Statistical analysis

The data were analyzed with GraphPad Prism 9.0 software. The default *p* < 0.05 was significantly different between groups. Multiple groups were analyzed by one-way Anova followed by Dunnett’s multiple comparisons test. Unpaired Student’s t-test was used for analysis between the two groups. Our experimental data were presented as mean ± SD.

## 3 Results

### 3.1 Targets of triptolide and OSCC

**FIGURE 1:**
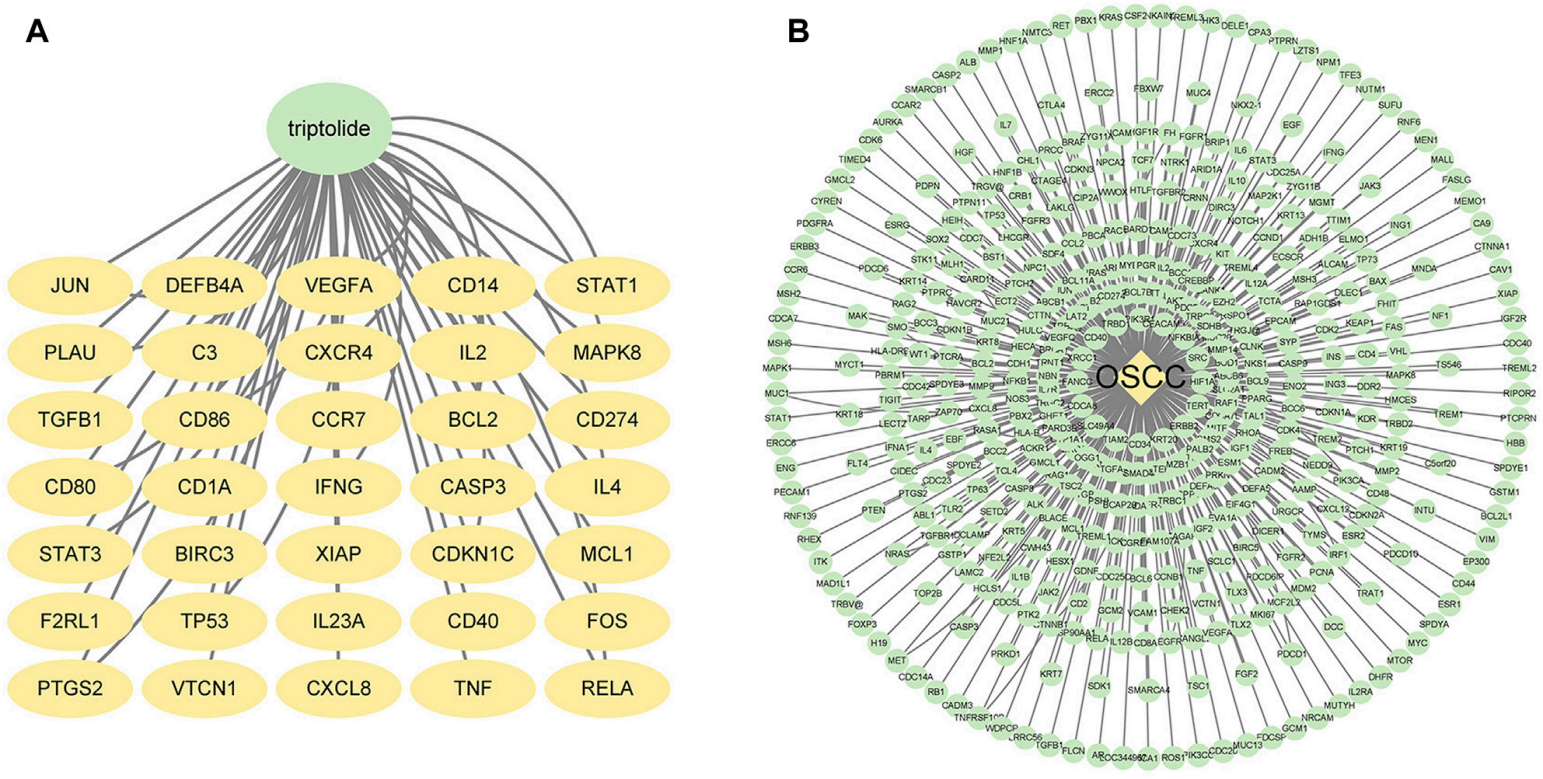
Potential targets of triptolide and OSCC. **(A)** Targets of triptolide. The green node represents triptolide, while yellow nodes show the related targets. **(B)** Targets of OSCC. The yellow node represents OSCC, while the green nodes show the related targets.

We searched the Swiss Target Prediction and TCMSP databases, and identified 35 targets predicted for triptolide. The drug–active compound–target network diagram reflects the correspondence between triptolide and the target (Figure 1A).

We also searched OMIM and GeneCards databases using the keyword “oral squamous cell carcinoma”. We found almost 5,500 related targets from GeneCards and selected the top 310 targets with relevance scores greater than 50.0134 (Figure 1B). We pooled these selected targets with 300 potential targets from OMIM, and 449 potential OSCC targets were obtained after removing the duplicates.

### 3.2 Core target screening of triptolide in OSCC treatment

**FIGURE 2:**
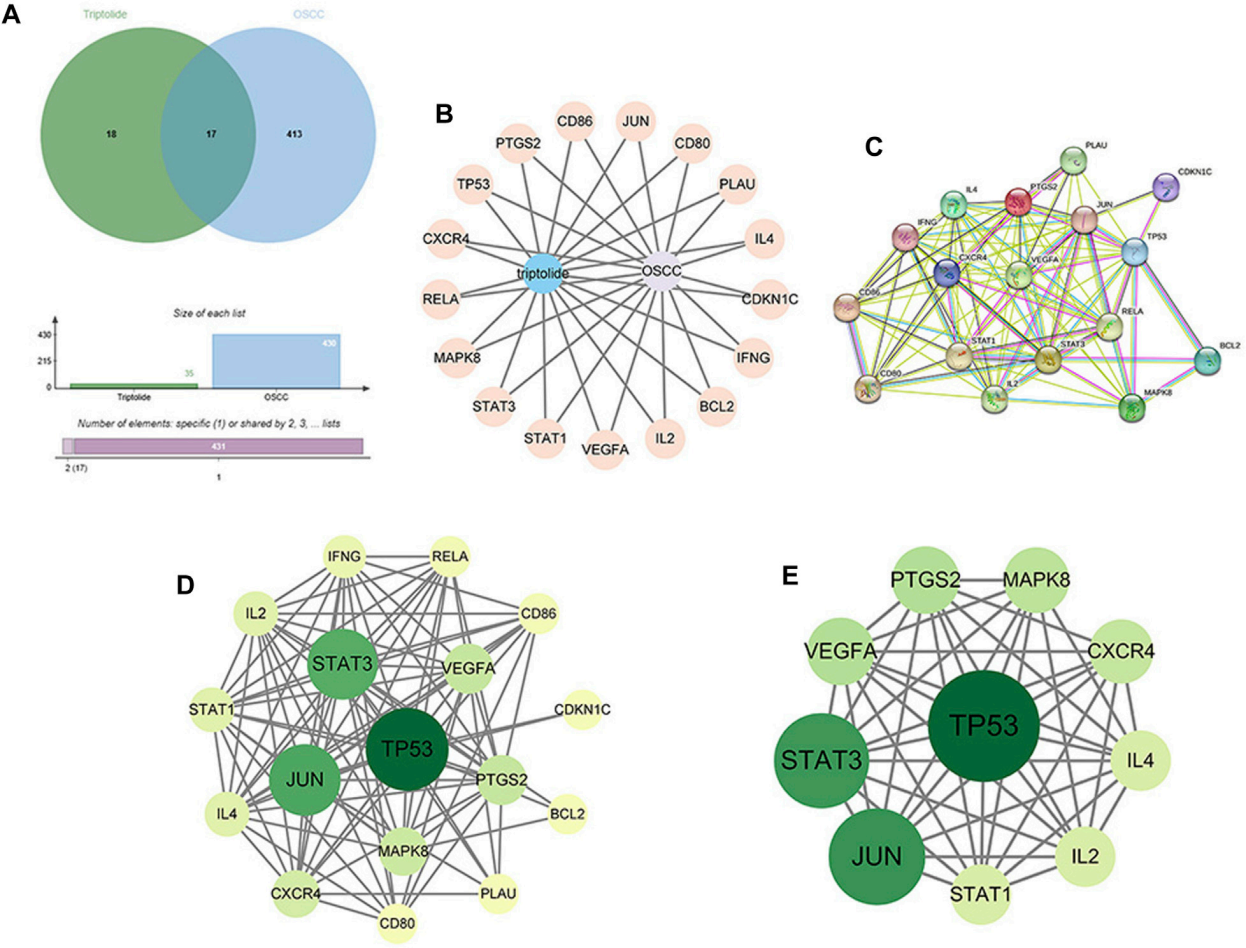
Construction of the PPI network and identification of the core targets. **(A)** Venn diagram of the intersection of 35 targets of triptolide and 430 targets of OSCC. **(B)** Construction of the compound-target-disease network (the blue nodes represent triptolide, and flesh-colored nodes represent potential therapeutic targets). **(C, D)** PPI network of potential core targets related to triptolide and OSCC. The nodes in darker color and a more significant size represented a higher degree. **(E)** Core target network, The top 10 core targets in the PPI network.

17 intersection targets were obtained after crossing the triptolide targets with OSCC targets (Figures 2A, B). The predicted 17 intersection targets were imported into the STRING database, and a protein interaction relationship map was generated (Figure 2C). The PPI network data were imported into Cytoscape to establish a network diagram of the interactions of the target proteins (Figure 2D). The core target network was obtained after an analysis of the topological characteristics, revealing a protein correlation between triptolide and OSCC (Figure 2E). The core target network contained 17 nodes and 93 edges. The yellow-to-green change in color of the nodes indicates a small-to-large betweenness value. A larger betweenness value suggests that more nodes interact with the gene. Based on the betweenness value, 10 key proteins were selected: PTGS2, STAT1, STAT3, VEGFA, CXCR4, TP53, Jun, MAPK8, IL-2, and IL-4.

### 3.3 GO and KEGG pathway enrichment analysis

**FIGURE 3:**
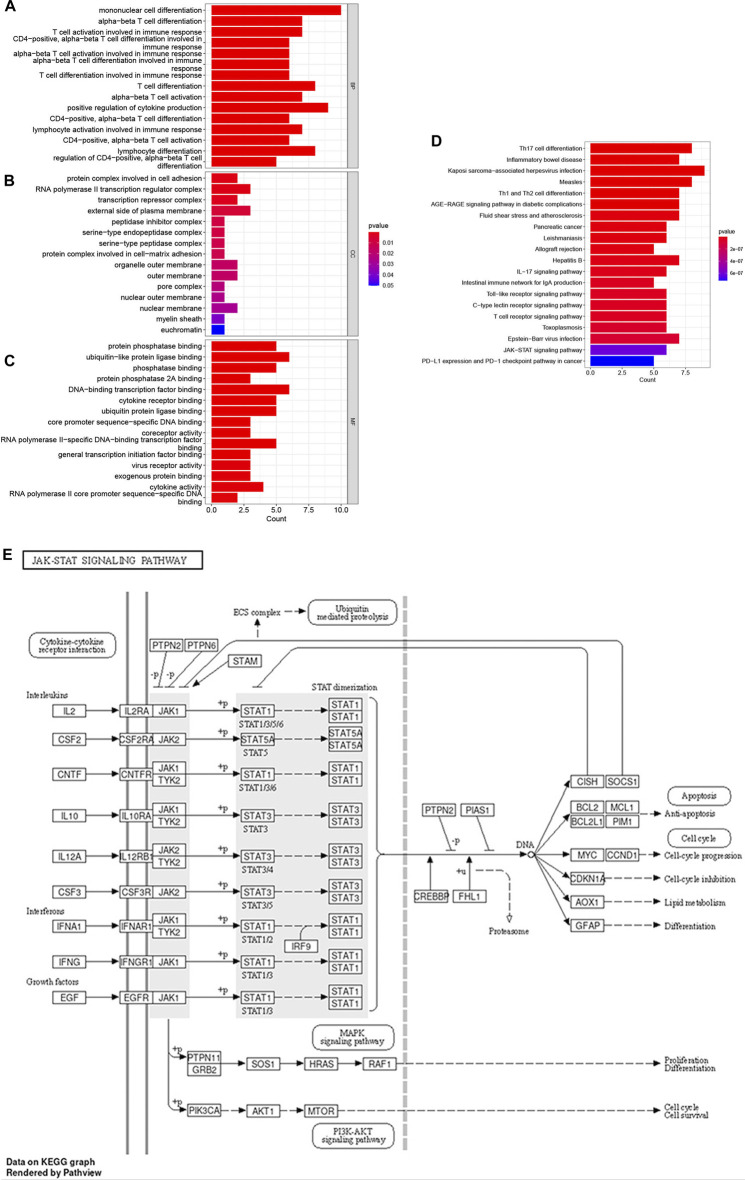
GO enrichment and KEGG pathway enrichment analysis of the 17 potential therapeutic targets. **(A–C)** GO enrichment analysis: the top 15 biological processes, molecular functions, and cell components, The color indicates the *p*-value. **(D)** Bubble chart of the top 20 KEGG pathways. The color indicates the *p*-value. **(E)** Relevant targets in the signaling pathway of triptolide and JAK-STAT.

R software was used to perform GO and KEGG pathway enrichment analyses of the 17 candidate target genes, which were calculated by applying the R package clusterProfiler (Supplementary Table S4). The GO analysis included biological processes (BPs), cellular components (CCs), and molecular functions (MFs). The BPs indicated that the targets are mainly associated with cell differentiation and immune cell activation such as mononuclear cell, lymphocyte and T cell differentiation, lymphocyte and T cell activation (Figures 3A–C). The results of KEGG analysis showed that the JAK–STAT signaling pathway plays an important role in OSCC (Figure 3D; Table 3). A prediction map of the JAK–STAT pathway associated with the targets of triptolide-treated OSCC is shown in Figure 3E.

**TABLE 3:** Top 20 pathways by KEGG analysis.

ID	Description	P-value	Count
hsa04659	Th17 cell differentiation	1.43E-11	8
hsa05321	Inflammatory bowel disease	2.43E-11	7
hsa05167	Kaposi sarcoma-associated herpesvirus infection	3.69E-11	9
hsa05162	Measles	1.11E-10	8
hsa04658	Th1 and Th2 cell differentiation	2.97E-10	7
hsa04933	AGE-RAGE signaling pathway in diabetic complications	5.37E-10	7
hsa05418	Fluid shear stress and atherosclerosis	5.49E-09	7
hsa05212	Pancreatic cancer	5.62E-09	6
hsa05140	Leishmaniasis	6.09E-09	6
hsa05330	Allograft rejection	9.25E-09	5
hsa05161	Hepatitis B	1.60E-08	7
hsa04657	IL-17 signaling pathway	2.05E-08	6
hsa04672	Intestinal immune network for IgA production	3.47E-08	5
hsa04620	Toll-like receptor signaling pathway	3.78E-08	6
hsa04625	C-type lectin receptor signaling pathway	3.78E-08	6
hsa04660	T cell receptor signaling pathway	3.78E-08	6
hsa05145	Toxoplasmosis	5.90E-08	6
hsa05169	Epstein-Barr virus infection	7.37E-08	7
hsa04630	JAK-STAT signaling pathway	6.15E-07	6
hsa05235	PD-L1 expression and PD-1 checkpoint pathway in cancer	7.19E-07	5

### 3.4 Molecular docking

**FIGURE 4:**
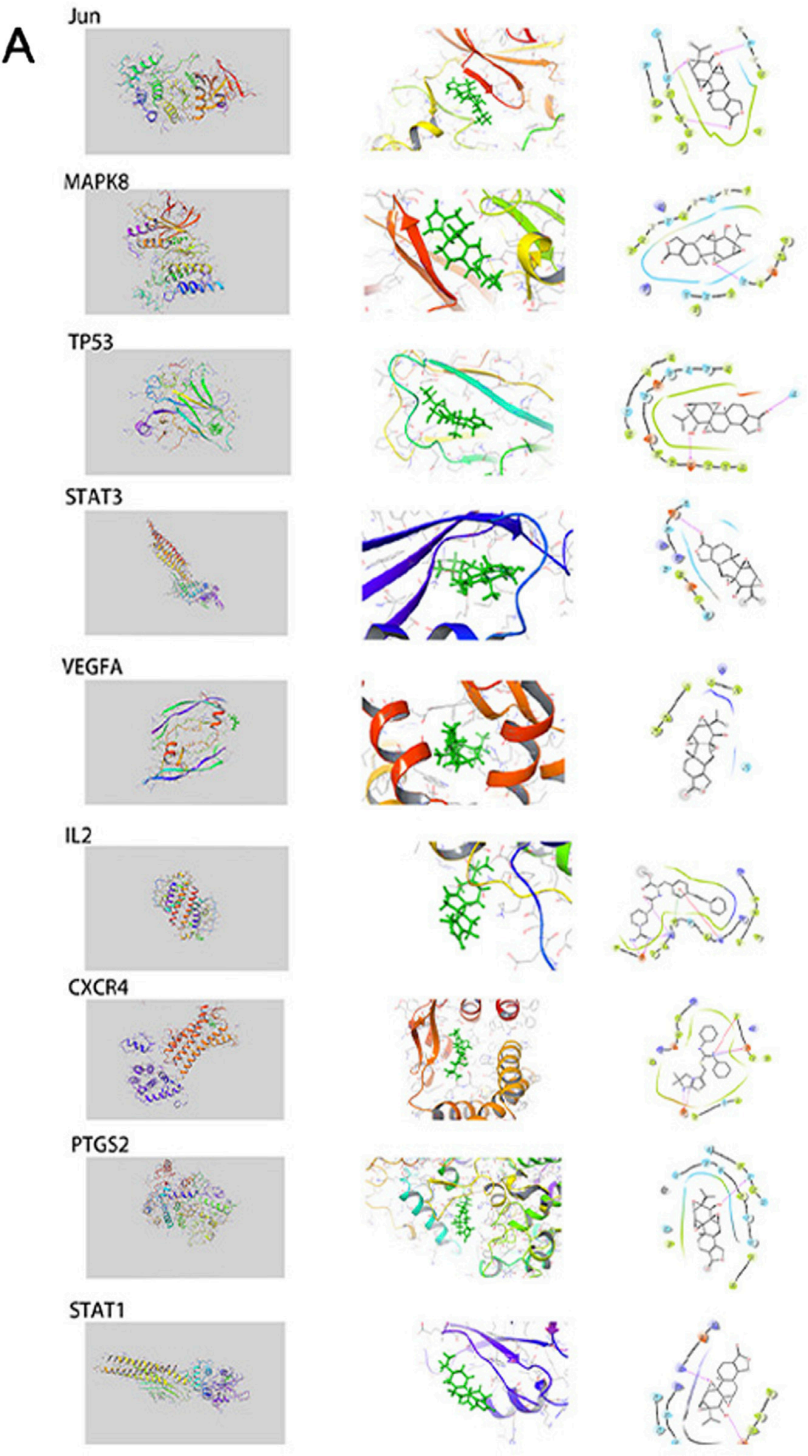
The interactions between triptolide and its core targets. This includes complete graph, partial graph and simplified graph.

To validate and visualize the predicted targets for triptolide in OSCC treatment, the target proteins and small molecules of triptolide were subjected to molecular docking simulations in Maestro. Binding energy is negatively correlated with the binding capacity between molecules and targets. Potential exists for triptolide binding to other predicted targets except for IL-4, and the docking scores of Jun, MAPK8, and TP53 were all less than −5 kcal/mol (Table 4), indicating a strong docking effect (Figure 4A). Jun, MAPK8 and TP53 are related to MAPK signaling pathway, which suggests that MAPK signaling pathway may play a central role in the treatment of oral squamous cell carcinoma by triptolide.

**TABLE 4:** Docking scores of triptolide and 10 key targets.

gene	PDB ID	Docking score
JUN	4y46	−5.977
MAPK8	3elj	−5.609
TP53	8dc6	−5.315
CXCR4	3oe8	−4.83
PTGS2	5fL9	−3.361
STAT1	7nuf	−2.968
STAT3	6njs	−2.426
VEGFA	6v7k	−1.959
IL2	1m48	−1.727
IL4		No matching options

### 3.5 Triptolide inhibited proliferation, migration, and expression of core target genes in OSCC

**FIGURE 5:**
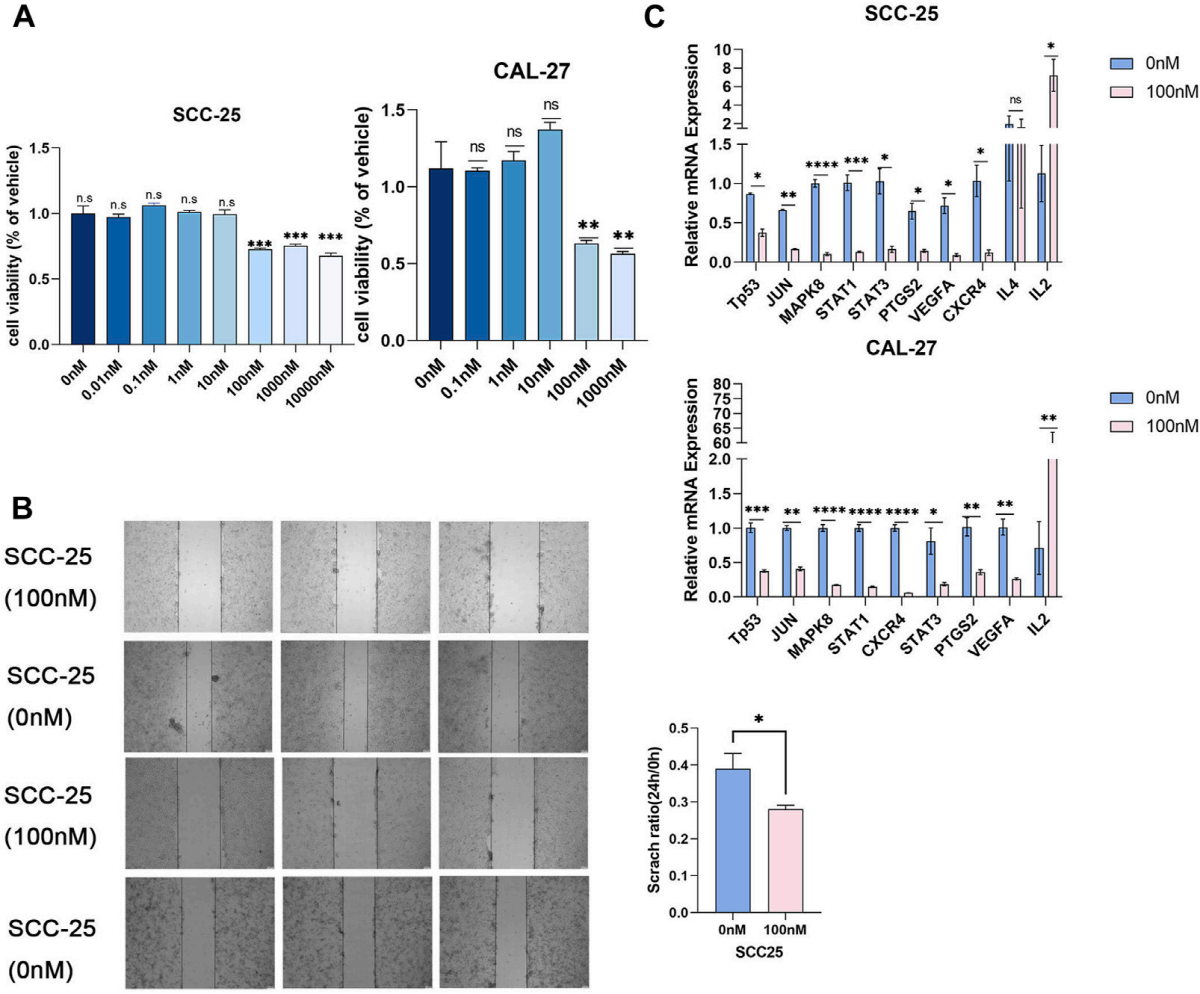
Triptolide inhibited OSCC cell proliferation. **(A)** The CCK8 assay was used to measure cell growth. Compared with the control group, *p < 0.05, **p < 0.01, ***p < 0.001. SCC-25 cells are left part and CAL-27 cells are right part. **(B)** The representative images of wound-healing assay in two groups. The statistics were expressed as Mean ± SEM, n = 3 (SCC-25 cells). Compared with the control group, *p < 0.05. The statistics were expressed as Mean ± SEM, n = 3 (CAL-27 cells). Compared with the control group, ***p < 0.001, **p < 0.01, *p < 0.05. **(C)** Triptolide inhibited core target expression in OSCC cells. Quantitative RT-PCR analysis of TP53, JUN, MAPK8, STAT1, STAT3, PTGS2, VEGFA, CXCR4, IL4 and IL2 mRNA expression. Compared with the control group, *p < 0.05, **p < 0.01.

We examined the proliferation and migration of SCC-25 and CAL-27 cells treated with different triptolide concentrations for 24 h (0, 0.01, 0.1, 1, 10, 100, 1,000 and 10000 nM). Cell proliferation was significantly suppressed at 100 nM triptolide; no significant difference was observed in cell proliferation at higher drug concentrations (Figure 5A). We compared the predicted gene expression levels in OSCC cells treated with triptolide. The mRNA levels of most of the predicted targets such as TP53, STAT1, and STAT3 were significantly downregulated, whereas IL-2 gene expression was increased. IL-4 expression did not change in SCC-25 cells and was not detected in CAL-27 cells (Figure 5B). The scratch wound-healing assay was performed with 100 nM triptolide as the effective concentration. Triptolide (100 nM) significantly inhibited wound closure, and the number of migrating cells in the control group was significantly higher than that in the triptolide-treated group (Figure 5C). The results of KEGG data and molecular docking indicated that the downregulated genes after triptolide treatment were mainly related to JAK-STAT signaling pathway and MAPK signaling pathway. Studies have shown that IL2 can inhibit tumor cell proliferation by influencing JAK-STAT signaling pathway through autocrine mode (Gotthardt et al., 2019). The results suggest that triptolide can inhibit JAK-STAT-related genes by increasing the expression of IL2 on the one hand, and inhibit MAPK signaling pathway related genes on the other hand, and jointly achieve the purpose of inhibiting cell growth and proliferation.

### 3.6 Triptolide suppressed tumors in xenograft mouse model

**FIGURE 6:**
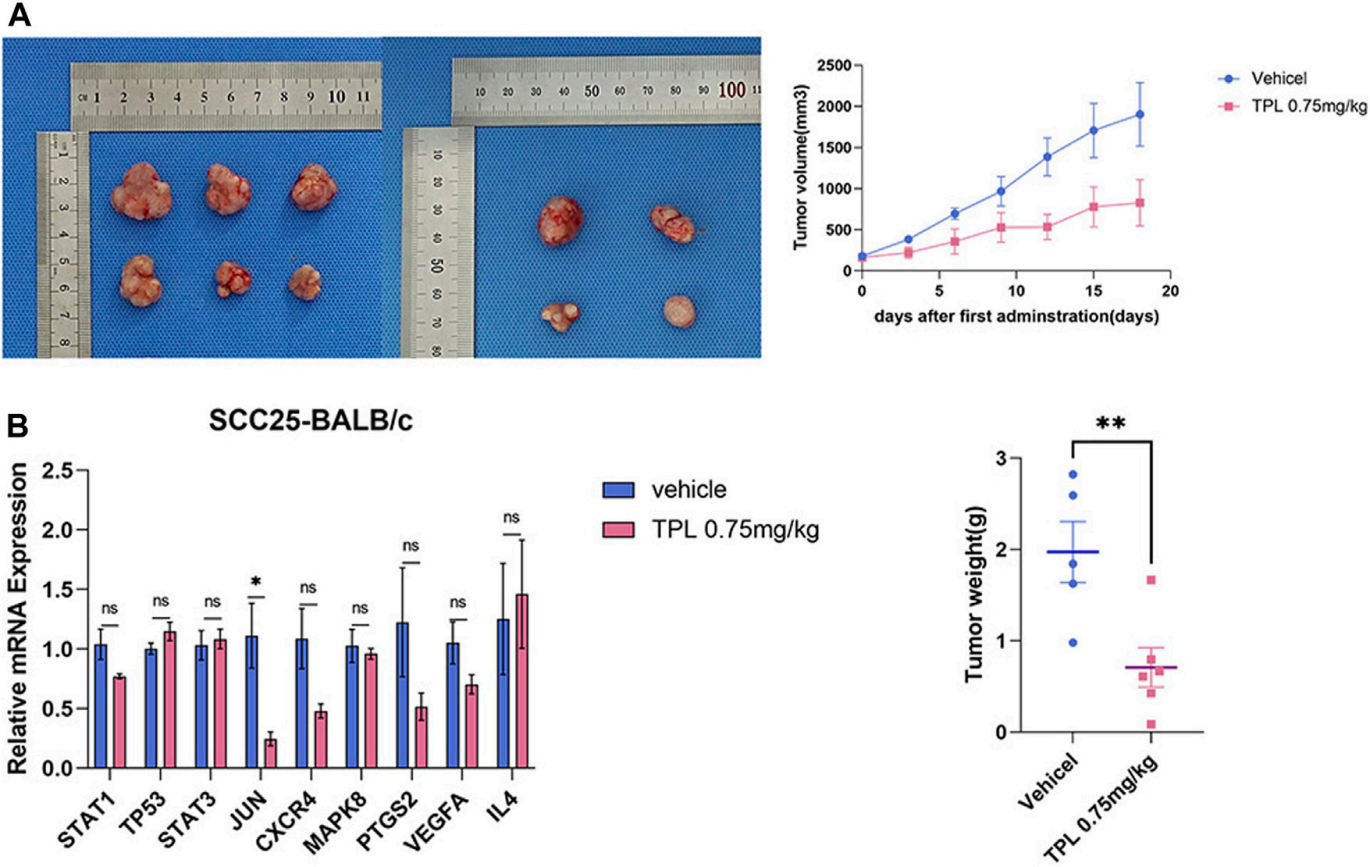
Combination of triptolide inhibits tumorigenicity of SCC-25 cell *in vivo*. **(A)**Growth curve and tumor weight of xenograft tumors in mice treated with single reagent of triptolide (0.75 mg/kg QD) with (*n* = 5/group). The macroscopic view of xenograft tumors at the endpoint of experiment. **(B)** Triptolide affects core target expression *in vivo*. Quantitative RT-PCR analysis of TP53, JUN, MAPK8, STAT1, STAT3, PTGS2, VEGFA, CXCR4 and IL4 mRNA expression. Compared with the control group, *p < 0.05, **p < 0.01.

SCC-25 cells were used to evaluate the inhibitory effects of triptolide *in vivo* in xenograft mouse model. The triptolide group showed a marked decrease in tumor volume and weight compared with the vehicle group (Figure 6A). The Jun mRNA expression decreased after triptolide treatment, whereas no significant difference was found in the expression of the other genes. However, the expressions of STAT1, CXCR4, PTGS2, and VEGFA tended to decrease (Figure 6B). The differences in the gene expressions between the *in vivo* and *in vitro* experiments may have been due to the indirect and limited effect of triptolide on cancer cells *in vivo* compared with that *in vitro*.

## 4 Discussion

**FIGURE 7:**
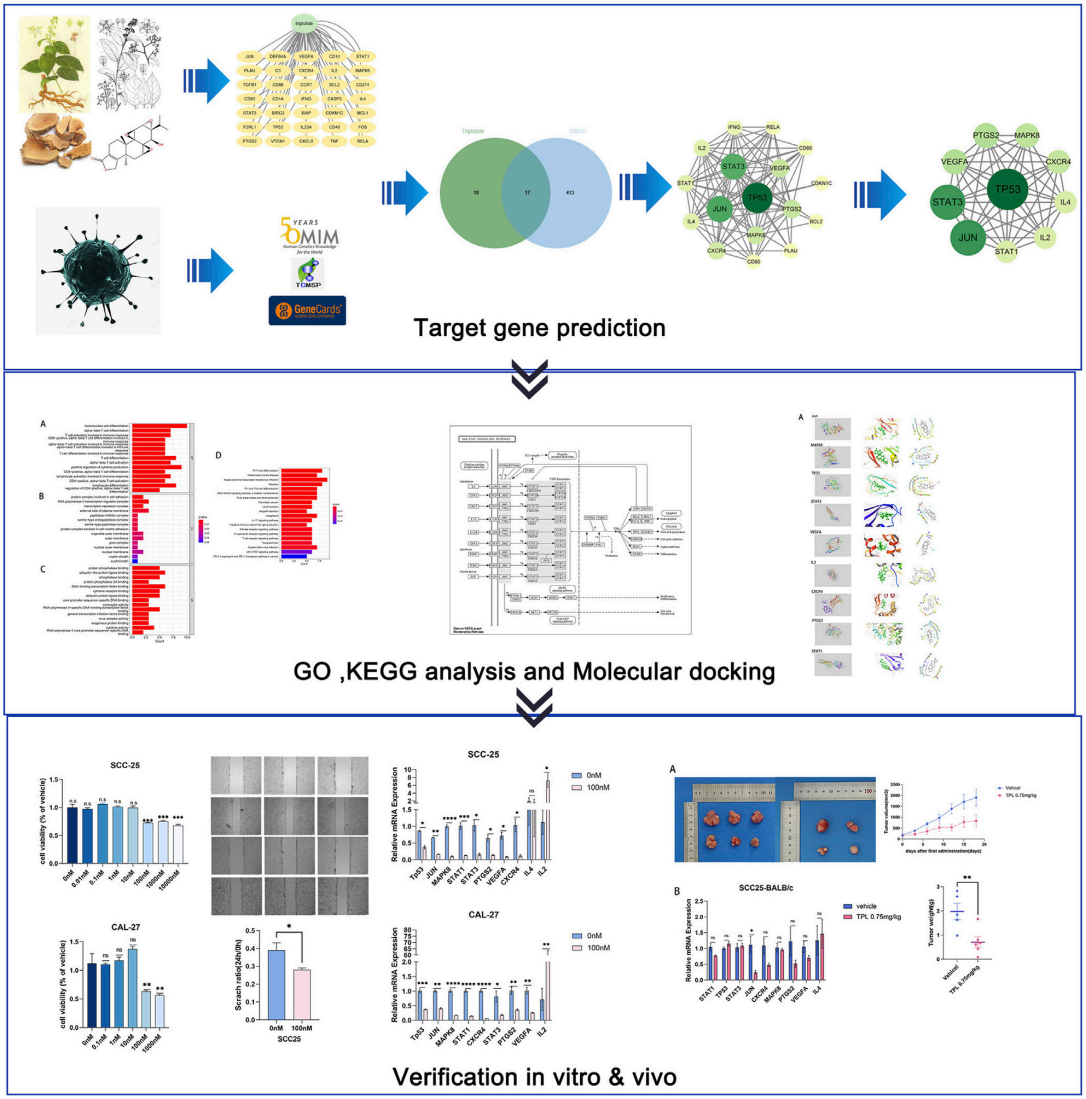
Mechanism of triptolide on oral squamous cell carcinoma.

In our study, we found 35 targets for triptolide and approximately 5,800 targets for OSCC. Of these, we selected 449 OSCC targets for subsequent analysis. We identified 17 nodes in the core PPI network after merging two networks. The BPs identified in the GO analysis that were enriched included the immune response and immune cell differentiation including T cells, mononuclear cells, and lymphocytes. KEGG analysis and *in vivo* experimental results showed that the JAK-STAT signaling pathway may play a key role in OSCC treatment (Figure 7).

The JAK–STAT signaling pathway is a pivotal aspect of cell function that mediates various processes required for homeostasis and development, including tissue repair, cell proliferation, inflammation, apoptosis, hematopoiesis, and immune fitness (Owen et al., 2019; Hu et al., 2021). Abnormal activation of the JAK–STAT signaling pathway is detected in various types of cancers, including head and neck, lung, pancreatic, breast, rectal, and prostate cancers (Xue et al., 2023). To date, researchers have found seven members of the STAT family (STATs 1–4, 5A, 5B, and 6); however, STAT proteins function differently in cancer (O’Shea et al., 2015). STAT3 acts as a critical player in OSCC occurrence and development (Jiang and Li, 2022). STAT3 increases cell proliferation and inhibits apoptosis in OSCC via several targets including Bcl-2, Bcl-xL, Survivin, and cyclin D1 (Deepak Roshan et al., 2018; Wei et al., 2022). Arecoline induces STAT3 expression, resulting in the upregulation of c-Myc expression (Chuerduangphui et al., 2018). STAT3 binds to the promoter region of Glut5 to enhance the transcription of Glut5 to increase the uptake and use of fructose for OSCC cell growth (Huang et al., 2022). JAK2/STAT3 signaling activates epiregulin-induced cancer-associated fibroblasts, which stimulates the epithelial–mesenchymal transition (EMT) of OSCC cells, which is necessary for migration and invasion (Wang et al., 2019). CCL18 promotes cell growth, metastasis, and EMT in OSCC through the JAK2/STAT3 signaling pathway (Jiang et al., 2020). Inhibition of HNF1A-SA1 expression suppresses the proliferation, migration, and EMT of OSCC cells through the Notch signaling pathway, whereas STAT3 promotes HNF1A-SA1 upregulation (Liu et al., 2019). CCL4 enhances Angpt2 and VEGF-C expressions via the MEK/ERK/STAT3 and JAK2/STAT3 pathways, respectively, to promote angiogenesis and lymphangiogenesis in OSCC, thereby increasing the propensity of metastasis (Lien et al., 2018; Lu et al., 2022). Additionally, STAT3 is associated with the chemosensitivity and radiosensitivity of OSCC cells (Chen et al., 2019; Jia et al., 2022). Fewer studies have been conducted on the association between STAT1 and OSCC than on the role of STAT3 in OSCC. FEN1 may directly or indirectly regulate HLA-DR and PD-L1 in OSCC via the IFN-γ/JAK/STAT1 pathway (Wang et al., 2023). Shan et al. reported that the downregulation of STAT1/BST2 axis inhibited OSCC cell growth through the AKT/ERK1/2 signaling pathway (Shan et al., 2023). Overall, above results demonstrate that the JAK/STAT signaling pathway is a potential molecular target and biomarker for OSCC.

Jun, TP53, STAT3, and MAPK8 (JNK1) were the predicted proteins in our findings, indicating that the MAPK signaling pathway is closely related to the treatment of OSCC. The results of several studies have supported the anticancer role of JNK alone or synergistically with downstream molecules in OSCC. Yang et al. reported that hispolon may activate the JNK pathway to induce caspase-dependent apoptosis (Yang et al., 2023). Chen et al. also demonstrated that triptolide can induce apoptosis of OSCC cell lines SCC-25 and OEC-M1 *in vivo* by activating caspase-25 and caspase-1 (Chen et al., 2009). The downregulation of B4GALNT1 can inhibit proliferation and growth in OSCC cells via the JNK pathway (Jing et al., 2020). Curcumin analogue HO-3867 affects cell growth in OSCC cells by triggering activated cysteine protease and PARP through the JNK1/2 pathway (Chen et al., 2022). Migration, invasion, adhesion, and EMT of OSCC cells are inhibited in a way that CHRDL1 affects the MAPK pathway (Wu et al., 2022). The pharmacological inhibition of JNK considerably inhibits cell invasion and the expressions of MMP-2 and MMP-9 in galectin-7-overexpressing OSCC cells (Guo and Li, 2017). However, some results support the tumorigenic role of JUK in OSCC. ICAM-1 expression is upregulated to increase OSCC cell migration due to IL-6 stimulation though JNK, Syk, and AP-1 signaling pathways (Chuang et al., 2014).

MAPK and JAK-STAT signal pathways do not exist independently, and there is a cross-talk association between them. JAK-STAT pathway is one of the highly conserved and structurally simple pathways in animals that transmit extracellular signals from transmembrane receptors to the nucleus with very few intermediate steps (Xin et al., 2020). JAK-STAT pathway is directly connected with a total of eight pathways, including MAPK signaling pathway (Zhao et al., 2021) and PI3K-Akt pathway (Habanjar et al., 2023), and plays a central role in tumors, closely related to cell differentiation, proliferation, apoptosis, immunity (Haftcheshmeh et al., 2022) and other functions, as also mentioned in the GO analysis above. JAK2-STAT3/MAPK-AP1 pathway supports OSCC survival, invasion, treatment resistance, and general immune function by regulating and regulating PD-L1 (Jha et al., 2023). SOCS1 can inhibit JAK/STAT and p44/42 MAPK pathways through overexpression, thus affecting tumorigenesis and growth of OSCC (Nakatani et al., 2022).

In summary, the results of this study indicate a potential tumor suppressor role of triptolide via JAK-STAT and MAPK signaling pathways, simultaneously down-regulating P53 downstream in OSCC treatment. However, discrepancies exist between the network pharmacology analysis and experimental results, which indicate that network pharmacology only provides a general direction for future study. The results need to be further verified through other experiments.

## 5 Conclusion

To sum up, triptolide is characterized by multiple components, multiple targets and multiple pathways. Triptolide has therapeutic effects on OSCC by down-regulating the expression of Jun, MAPK8, TP53, STAT3, VEGFA, IL2, CXCR4, PTGS2, and inhibiting cell growth. The molecular mechanism of triptolide in the treatment of OSCC is deeply explored to provide a new approach and provide a new idea for the study of targeted therapeutic mechanisms.

## Data Availability

The original contributions presented in the study are included in the article/Supplementary Materials, further inquiries can be directed to the corresponding authors.
