# Supplementary material for: Network pharmacology and experiment validation investigate the potential mechanism of triptolide in oral squamous cell carcinoma
Source: Front Pharmacol. 2024 Jan 8;14:1302059. doi: 10.3389/fphar.2023.1302059 (PMC10800448; doi:10.3389/fphar.2023.1302059)
Supplement: Supplementary file 2 [file Table3.DOCX]

| 基因 | Forward Primer | Reverse Primer |
| --- | --- | --- |
| PTGS2 | ATTGTACCCGGACAGGATTCTATG | TTTGGAGTGGGTTTCAGAAATAATT |
| STAT3 | GAGGACTGAGCATCGAGCA | CATGTGATCTGACACCCTGAA |
| VEGFA | CTGGAGCGTGTACGTTGGT | GTTTAACTCAAGCTGCCTCGC |
| JUN | GCCTCAGACAGTGCCCGAGAT | GTTTAAGCTGTGCCACCTGTTCC |
| TP53 | GCTGAGTATCTGGACGACAGG | AGCGTGATGATGGTAAGGATG |
| MAPK8 | TGTGTGGAATCAAGCACCTTC | AGGCGTCATCATAAAACTCGTTC |
| STAT1 | TCCGTTTTCATGACCTCCTG | TGAATATTCCCCGACTGAGC |
| IL2 | CAAACCTCTGGAGGAAGTGC | AATGGTTGCTGTCTCATCAGC |
| IL4 | GTCTCACCTCCCAACTGCTT | CTTGGAGGCAGCAAAGATGT |
| CXCR4 | GGAGGGGATCAGTATATACA | GAAGATGATGGAGTAGATGG |
