# Supplementary material for: Network pharmacology and experiment validation investigate the potential mechanism of triptolide in oral squamous cell carcinoma
Source: Front Pharmacol. 2024 Jan 8;14:1302059. doi: 10.3389/fphar.2023.1302059 (PMC10800448; doi:10.3389/fphar.2023.1302059)
Supplement: Supplementary file 4 [file Table4.DOCX]

getwd()

setwd('D:\\hpy\\shengxinkouqiang\\genejiaoji\\PPIhongsu\\Rfenxi')

if (!require("BiocManager", quietly = TRUE))

install.packages("BiocManager")

BiocManager::install("org.Hs.eg.db")

if (!require("BiocManager", quietly = TRUE))

install.packages("BiocManager")

BiocManager::install("clusterProfiler")

detach("packages:enrichplot")

rm(list=ls())

library("org.Hs.eg.db")

library("clusterProfiler")

library("enrichplot")

library("ggplot2")

library("ggnewscale")

library("enrichplot")

library("DOSE")

library(stringr)

pvalueFilter=0.05

qvalueFilter=1

showNum=15

rt=read.table("target.txt",sep="\t",check.names=F,header=F)

genes=as.vector(rt[,1])

entrezIDs <- mget(genes, org.Hs.egSYMBOL2EG, ifnotfound=NA)

entrezIDs <- as.character(entrezIDs)

rt=cbind(rt,entrezID=entrezIDs)

colnames(rt)=c("symbol","entrezID")

rt=rt[is.na(rt[,"entrezID"])==F,]

gene=rt$entrezID

gene=unique(gene)

colorSel="qvalue"

if(qvalueFilter>0.05){

colorSel="pvalue"

}

write.csv(BP)

kk=enrichGO(gene = gene,OrgDb = org.Hs.eg.db, pvalueCutoff =1, qvalueCutoff = 1, ont="all", readable =T)

GO=as.data.frame(kk)

GO=GO[(GO$pvalue<pvalueFilter & GO$qvalue<qvalueFilter),]

write.table(GO,file="GO.xls",sep="\t",quote=F,row.names = F)

if(nrow(GO)<30){

showNum=nrow(GO)

}

pdf(file="GO_barplot.pdf",width = 9,height =12)

bar=barplot(kk, drop = TRUE, showCategory =showNum,split="ONTOLOGY",color = colorSel) + facet_grid(ONTOLOGY~., scale='free')+scale_y_discrete(labels=function(x) stringr::str_wrap(x, width=60))

print(bar)

dev.off()

pdf(file="GO_bubble.pdf",width = 9,height =7)

bub=dotplot(kk,showCategory = showNum, orderBy = "GeneRatio",split="ONTOLOGY", color = colorSel) + facet_grid(ONTOLOGY~., scale='free')+scale_y_discrete(labels=function(x) stringr::str_wrap(x, width=60))

print(bub)

dev.off()

pdf(file="GO_cnet.pdf",width = 10,height = 5)

af=setReadable(kk, 'org.Hs.eg.db', 'ENTREZID')

cnetplot(kk, showCategory = 10, categorySize="pvalue",circular = TRUE,colorEdge = TRUE,cex_label_category=0.65,cex_label_gene=0.6)

dev.off()

pdf(file="GO_net.pdf",width = 9,height = 7)

x2 <- pairwise_termsim(kk)

emapplot(x2,showCategory = 20,cex.params = list(category_label = 0.65),color = "pvalue",layout.params = list(layout = "nicely"))

dev.off()

library(dplyr)

BP<-filter(GO,GO$ONTOLOGY =="BP")

go_enrich_df$type_order = factor(rev(as.integer(rownames(go_enrich_df))),labels=rev(go_enrich_df$Description))

ggplot(data = go_enrich_df, mapping = aes(x=type_order,y=log10.pValue)) +

geom_point(aes(size=Count,color=log10.pValue))+

scale_color_gradient(low="blue",high ="red")+

ylab("pvalue") +

xlab("GO term") +

facet_grid(.~Ont , scale="free") +

theme(axis.text.x = element_text(angle = 90 , hjust = 1))
